# The Culture of E-Arabs

**DOI:** 10.3390/jintelligence11010007

**Published:** 2022-12-28

**Authors:** Abdulrahman Essa Al Lily, Abdelrahim Fathy Ismail, Fathi Mohammed Abunasser, Rafdan Hassan Alhajhoj Alqahtani, Firass Al-Lami, AlJohara Fahad Al Saud

**Affiliations:** 1 Department of Curriculum and Teaching Methods, College of Education, King Faisal University, Al Ahsa 31982, Saudi Arabia; 2 College of Education, Assiut University, Assiut 71515, Egypt; 3 Educational Editing Department, Scientific Journal of King Faisal University, Al Ahsa 31982, Saudi Arabia; 4 Educational Foundation and Administration Department, College of Education, Sultan Qaboos University, Seeb 123, Oman; 5 Deanship of Information Technology, King Faisal University, Al Ahsa 31982, Saudi Arabia; 6 Education and Psychology Department, College of Education, King Faisal University, Al Ahsa 31982, Saudi Arabia; 7 Early Childhood Education Department, College of Education, King Saud University, Riyadh 11362, Saudi Arabia

**Keywords:** Arab sociology, digital sociology, digital anthropology, Twitter, Arab social media

## Abstract

This article scrutinises the linkage between ethnicity and people’s behaviour on Twitter. It examines how offline culture manifests itself online among Arabs. The article draws upon the literature to identify the offline ethnic characteristics of Arabs, and through interviews with and observations of Arab social media users, discovers their online ethnic characteristics. It then compares these online and offline characteristics and, through this comparison, finds that offline culture has been enacted online among Arabs, sustaining expressions of generosity, religiosity, traditionalism, female privacy, over-flattery, collectivism, tribalism, pan-Arabism, and social contracts; however, in other ways, offline culture has been counteracted online, which has led to the destabilisation of power relations between genders, elites and non-elites, and majorities and minorities. A further finding is that online culture has been enacted offline among Arabs in that online performance has exerted influence over offline ethnic identity expectations. In short, there are three main findings: offline culture has been enacted online, offline culture has been counteracted online, and online culture has been enacted offline. The take-home finding of this study is the existence of ‘e-ethnic culture’, that is, although ethnic activity online tends to be based on and reinforces offline realities and may alter offline realities as well, not all online performances have roots offline.

## 1. Literature Review

### 1.1. Theoretical Background

This manuscript investigates the interplay between ethnic identity and cyber-space. At the intersection of ethnic identity development and the Internet (Nakamura 2013), theorists are in dispute about whether ethnicity exerts a significant influence on people in online settings. What follows is a summary of the relevant literature.

On one side of the debate, academics deny, elide, or repress the existence of ethnic dynamics online (cf. Zagboush 2014). They refuse to recognise the online public sphere as entailing a ‘culture’ (Zagboush 2014) because, in their view, culture is a stable entity, which, while exhibiting gradual change over an extended period of time, is incompatible with the changeable dynamics of online spaces, which are inherent due to the rapid nature of digital technological development (cf. Winner 1978). Moreover, they argue that the disassociation of online communication from spatiality and materiality does not correspond to the conventional conceptualisation of what culture is, denying the online environment the capacity to house cultures (Dutton et al. 2014). What the online environment lacks in spatiality, they contend, it also lacks in temporality: a culture has to be rooted, and since online communication is a recent phenomenon and thus lacks historicity, it cannot be acknowledged as having as yet evolved into a culture (da Rosa et al. 2001). Some theorists take their analysis a step further, reasoning that ethnic unity has been eroded due to the substantial intensification of cross-cultural mixing, leading to many similarities (cf. Feenberg 1999). This erosion of ethnic expression online means that, as a variable, it is ‘nothing but […] abstract’ (Dahan and Sheffer 2001, p. 88).

On the opposing side, anthropologists put forward the argument that offline ethnic ties do not simply dissolve in online milieux (Matei and Ball-Rokeach 2001) but, rather, are moulded by the Internet into a new form, ‘neoethnicity’ (Poster 1998, p. 184), engendering an ethnic revival. According to these anthropologists, the scholarly examination of ethnicities should recognise their self-definitions, self-construction, self-organisation, and self-assertiveness in the digital world because, in their eyes, ethnic consciousness is ‘present everywhere on the Internet’ (Diamandaki 2003, p. 30). To accept this position is to discard the idea of the Internet being space where ethnicity has been eliminated. The Internet may, thus, be better described as an ethnicity-enabling domain (Parker and Song 2006). In line with this stance, Poepsel (2018) proposes that owing to their attachment to their offline settings, individuals will find ways to mould their offline ethnic culture to fit into an electronic shape. Wilson and Peterson (2002) share a similar view that people’s physical-world ethnic traditions, norms, and values may direct and determine their activity online, implying a situation of ‘offline rules, online tools’, i.e., offline cultural traits that guide online behaviour (Clauser 2001). Howard et al. (2001), likewise, put forward the argument that, through online communication, one has the power to mould a digital environment, so much so that it becomes largely akin to the environment of one’s offline ethnic culture. 

### 1.2. Cultural Background

Philosophers call for investigating ‘the type of relationships that exist between the “real” and “virtual” ethnic subject’ (Marotta 2011, p. 540). They encourage ancillary initiatives to examine how the world’s cultures manifest themselves online among ethnic groups (Karakusheva 2016). The current study seeks to participate in this task, though with a particular focus on Arab ethnicity. ‘Arabs’ are regarded here as those who speak Arabic and are of Arab origin, that is, those with roots extending back to the tribes from Arabia. Arab society is directed, educated and, at times, controlled to conform to cultural values and social norms. It is a collective society driven by Arab unity and pan-Arabism. This has made the norms and values of Arab society, at least in the offline world, readily recognisable and straightforward to document. The authors were curious whether, in the online domain, the norms and values of Arabs are similarly transparent. They were keen to document the behaviour of Arabs when they go online to uncover whether Arab society maintains cultural identity, ethnic homogeneity, collectivity, and unity in online settings.

Previous writings have addressed online ethnicity both theoretically and in general terms (Tynes et al. 2008); the current study stands apart by concentrating on one particular ethnic group and empirically documenting its online configurations. Similarly, many studies on online ethnicity principally employ structural, psychological, and political perspectives (e.g., enquiring into how ethnic minorities in offline settings behave in online settings) (Maurer-Fazio 2012), while the current study, in contrast, adopts anthropological and sociological lenses. Online inter-ethnic contacts have been detailed by other publications (see, for instance, Dekker et al. 2015); it is, rather, intra-ethnic contacts (i.e., among e-Arabs) that interest the current authors. Finally, what makes this study unique among studies in the literature is its concentration on the untheorised online ethnicity of Arabs. 

To properly examine how culture manifests itself online among Arabs, it is necessary to first examine the literature to identify the offline ethnic characteristics of Arabs and, through research, to examine how these characteristics manifest themselves online. A systematic qualitative review of 249 publications was, thus, conducted to identify the offline ethnic characteristics of Arabs, thereby establishing the cultural context of the study and helping prepare the reader to see, later in the Findings sections, how such offline ethnic characteristics have been enacted or counteracted online. The review points to some key offline characteristics of Arabs that later will help interpret the findings. It is hoped that this review guides the reader through what Arab culture is (explained here in the literature review) and how Arab culture is enacted or counteracted online (discussed in the Findings sections).

The reviewed publications include books, book chapters, articles, master’s dissertations, and Ph.D. theses (e.g., Weisfeld 1990). Some of these publications are dedicated to the description of Arabs, whereas others describe Arabs only in some sections (e.g., Phillips 2013). The sources of data were accessed in two main ways. The first was traditional and physical: visiting libraries, bookstores, publishers (in Egypt and Jordan), and book fairs (e.g., those held in Riyadh and Bahrain) and collecting hard copies of relevant publications and manually going through them. The second way was modern and digital: searching relevant keywords in enormous Arab catalogues and search engines (e.g., Dar Almandumah and the Saudi Digital Library). 

A search strategy was developed to determine eligible publications and those to be excluded. An effort was made to broaden the search in order to maximise the retrieval of relevant results. That is to say, we explored not only academic but also non-academic literature, such as newspaper articles, novels, and poems. Non-academic literature was not the main informant and was used merely to enrich the results of the review. The inclusion of non-academic literature was, nonetheless, considered important because Arabs’ documentation of their culture in non-academic literature exceeds their efforts to do so in academic literature, which remains limited and modest. The search strategy entailed using keywords, synonyms, antonyms, and index terms. It also involved checking the references of the relevant publications that were found and exploring the publications of writers about Arab culture.

The review has singled out specific offline ethnic characteristics of Arabs that later shall assist with the interpretation of the findings. For example, offline, a woman may conceal her identity by wearing a face covering (Al-Shehab 2010). Another typical characteristic is that pan-Arabism is sustained, whereby Arabs support one another (cf. Valassopoulos 2013). Likewise, substantial familial and tribal support (be it deserved or undeserved) is normally lent by extended relatives to each other (Thompson 2019). It is common practice that one accuses others of corruption, untrustworthiness, and the breaching of the social contract between citizens and governments (Hanfi 2001). One commonly criticises others’ tribes and others’ culturally unacceptable deeds (see Al-Jabri 2011). One usually sustains a sense of collectivism, intervenes in others’ affairs, and gives them advice on their private, social, and professional lives (Buda and Elsayed-Elkhouly 1998). Discrimination based on race, nationality, tribe, ethnicity, religious schools of thought, pronunciation, dialects, and sects is prevalent (Al-Omari 2008). One habitually seeks to increase one’s influence by such acts as having multiple children, ensuring social presence, or giving gifts in exchange for loyalty, subordination, and power (Dwairy et al. 2006). Through curricula vitae or daily social or professional discourses and the like, there is customary exaggeration of achievements, falsification of knowledgeability, and praise of oneself, showing a sense of common narcissism (Al Lily 2018). An additional relevant characteristic is that one may trigger societal sympathy by putting on a show of illness, sharing news of death, or showing generosity (Al Zahrani 2002). This research aims to explore the extent to which each of these characteristics has been transferred to online settings.

This section has shed light on the theoretical disagreement within the literature over the existence of ethnic dynamics online, which is relevant given that this study lies at the crossroad of ethnicity and the Internet. The section has also drawn upon the existing literature to identify the offline ethnic characteristics of Arabs so as to build a cultural background for the study. The subsequent section demonstrates how Arab users of social media were interviewed and observed to identify the online ethnic characteristics of Arabs. The section that follows, Findings, compares these online and offline characteristics, and the final section, Concluding Remarks, relies on this comparison to discuss the power dynamics between online and offline domains in relation to ethnic behaviour and activity.

## 2. Methodology

### 2.1. Rationales

This article examines the relationship between ethnicity and the online domain, investigating whether the offline ethnic characteristics of Arabs are transferred to online spaces. In other words, this is an examination of two entities that appear, at least at first, to be incompatible: a traditional entity (Arab ethnicity) and a modern entity (the Internet). This apparent incompatibility between the two entities appears to be worthy of academic investigation (cf. Cohen-Mor 2018). Another rationale for this study is the authors’ academic interest in recording the interaction between online and offline social (here, ethnic) configurations. Studies on this interaction are limited, especially in the Arab context. One reason for choosing the Arab ethnicity was that the authors are Arabs and, therefore, insiders in the context under study, granting them privilege in terms of accessing Arab literature and understanding its cultural domain. Another reason was that Arab ethnicity exists as a force that shapes the public and private aspects of Arab society, and yet the online domain also exists as a force that controls contemporary society to a large degree; hence, the authors were interested in enquiring into the political interaction between these two forces, examining how a struggle may exist between them, as they simultaneously shape and are shaped by one another. The authors were also keen to observe whether their fellow Arabs represent or discard their ethnic backgrounds when online and to learn the extent to which Arab ethnicity manifests itself in the online world, if at all.

### 2.2. Data Collection

The authors worked on this project from 2020 to 2021. Four methods of data collection were used to address the research question of how and to what extent offline culture manifests itself online among Arabs.

The first was a participant observation of Arabs on Twitter. The researchers spent, on average, one hour per day over a period of 12 months on Twitter. They observed and participated in interactions among Arab tweeters, mapping their tweets, retweets, likes, comments, pictures, profile descriptions, followers, and following accounts.

The second was a non-participant observation of individual tweeters’ activity on Twitter. The researchers made a public announcement on Twitter that invited Arab tweeters to be subjects in individual observations. While navigating Twitter, they would share their computer screens and thoughts with the researchers. Thirty-four people accepted the invitation. 

The third method of data collection was collective interviewing. The researchers made a public announcement on Twitter that invited Arabs to be subjects in focus groups. Seven focus groups were formed. Each group consisted of five respondents, except for one group with six, totalling 36 respondents.

The fourth method was individual interviewing. A public announcement was made on Twitter that invited Arabs to participate in one-on-one interviews. Unstructured 30-min individual interviews were conducted with 38 individuals.

This study seeks richness and depth (not representation), that is, developing a rich and profound conceptual framework covering the most diverse and comprehensive views possible. Hence, a maximum variation sampling technique was applied to determine whether to admit a participant to the project. Maximum variation sampling (also known as maximum diversity sampling, maximum heterogeneity sampling, and heterogeneous sampling) is a purposive sampling method whereby researchers seek to collect data from the widest range of attributes, behaviours, experiences, incidents, qualities, and angles to gain a more holistic view of a specific matter (Crabtree 1999). For this sampling, units (here, individuals) are supposed to be tenaciously chosen for heterogeneity, that is, to be as varied as possible in terms of demographic variables. 

The maximisation of diversity and variance among community members meant carefully choosing study participants on the basis of various demographic variables: gender, nationality, sector (public and private sectors), and qualification (no formal qualification, primary, middle, or secondary school, undergraduate or master’s student, and assistant, associate, or full professor). Other variables included background (urban, rural, tribal Bedouin, and tribal non-Bedouin), economic class, years of Twitter membership (3–4, 5–6, 7–8, and 9 or more) and age (18–19, 20–29, 30–39, 40–49, 50–59, 60–69, and 70 or more). If there was more than one person with similar demographic characteristics, only one of them was admitted to the project. Increasingly more people were approached until at least one person was found in most of the variables.

Invitees to the research project were informed that only Arabs were eligible to participate in the study. There were two eligibility criteria: speaking Arabic and of Arab origin. Notably, whether one met these criteria was a matter of self-perception, which was intentional on the part of the authors to allow anyone who perceived themselves as an Arab to participate in the study. The informed consent sheet stated that the operational definition of ‘an Arab’ denotes a social and cultural mosaic, whereby one identifies oneself as an Arab regardless of religion, place of birth, residence, or accent.

Although the offline public domain of Arab society is male dominated, its online counterpart witnesses almost equal participation. This meant that the collective observations were able to document the behaviour of men and women almost equally. In the individual observations, focus groups and individual interviews, the authors ensured as close to equal participation of men and women as possible. The mean age range of the participants was 20s to 30s.

### 2.3. Data Analysis

The data were analysed thematically by breaking them down into marks and micro, meso, and macro visions (see Table 1). First, each relevant statement by the interviewees and each note from the observations was given a ‘mark’ (a keyword or short phrase) that indicated its essential meaning. Second, marks of the same kind were then gathered into ‘micro visions’, thereby creating initial impressions and taking early steps towards making sense of the data. Third, micro visions of the same type were subsequently grouped to form medium-level, clearer ‘meso visions’, thus taking moderate steps towards an understanding of the data. Fourth, the meso visions were assembled to eventually build comprehensive ‘macro visions’, which constituted the final step towards the overall process of making sense of the data.

This inductive process also entailed a deductive approach, as the emerging visions fed and were fed by the literature review (i.e., the Cultural Background sub-section). All the authors worked together to collect the data. The first author (the project manager) was entrusted with the task of analysing the data alone using a ‘codebook’ (McAlister et al. 2017) that the authors had collectively composed as a guideline for the analyst. Any research project consists of various tasks that are distributed among the research team; in the current project, the first author was assigned the task of analysing the data, whereas the other authors acted as auditors over the analytical work once it had been completed by the first author. 

In parentheses at the end of finding statements, Arabic numerals refer to the observers or interviewees. There are also letters inside the parentheses, where ‘OC’ refers to the observers of the collective, ‘OI’ to the observers of individuals, ‘I’ to the individual interviewees, and ‘F’ to the focus groups. For instance, a sentence followed by (OC-4) refers to a note of the fourth observer of the collective, (OI-6) refers to a note of the sixth individual observer, (I-5) refers to a statement of the fifth individual interviewee, and (F-3) refers to a statement of the third focus group.

In line with good academic practice, especially given the qualitative nature of the research, this paragraph describes the research team. The team consisted of Arabs of both genders, with working-class, middle-class, and upper-class backgrounds, who were in their 20s, 30s, and 50s, were from different Arab countries, and had lived in various Arab regions, including Saudi Arabia, Jordan, Egypt, and Oman. They had held different managerial positions, including dean of research, head of department, research consultant for a large higher education institution, director of a national research centre, and member of a national consultative assembly, and they had research interests that lay at the intersection of sociology, anthropology, education, management, and information technology.

## 3. Findings I (Offline Culture Being Enacted Online)

This section demonstrates how offline culture may be enacted online among Arabs in terms of online ethnic identity behaviour aligning with offline ethnic identity expectations.

### 3.1. E-Arabs Alongside Others

#### 3.1.1. E-Arabs Empowering Others

The data analysis highlights multifaceted factors related to the Internet-based empowerment of individual Arabs. For example, online, one may conceal one’s identity (mark: e-identity). A large number of female Arab tweeters conceal their identities by using a photo of flowers or a celebrity as their profile picture (OC-1). Such an act of blocking and occluding their images and likenesses in this way can be understood as a digital manifestation of the offline female veil. That is, offline, some women also conceal their identity by wearing a face covering (Almunajjed 1997).

#### 3.1.2. E-Arabs Honouring the Present

An especially striking finding pertains to e-Arabs’ various actions in honouring the present. For instance, online, relatives support one another (mark: e-familialism). When one tweets praise of one’s family or tribe, one receives many comments and followers from familial or tribal extended relatives and associates (OI-26). It is ordinary practice for someone involved with a poll to implore relatives to vote for them (OI-30). Pride in blood relationships is an inherent trait of Arab tweeters (OC-1). This online characteristic is prevalent to a similar extent offline, as expressed in the Arab maxim that ‘I am with my brother against my cousin, and my cousin and I are against the stranger’. Another online characteristic of e-Arabs is that online, loyalty is shown to important individuals (mark: e-flattery). Many Arab tweeters follow important people and extensively and insincerely like and retweet their tweets. They also write poems about them, send fan messages, and write complimentary tweets to them. These are viewed as effective strategies to integrate themselves with important people to gain favours from them. This online characteristic is prevalent to a similar extent offline, where an established hierarchy of social importance is sustained (Mauger 1988). Additionally, Arab literature is full of narratives, novels, and poems that show important people granting favours in return for praise (Al-Jabri 2011).

Homophily is the concept that individuals prefer to connect with people of similar traits, be they ethnicity, religion, nationality, gender, age, profession, or class (Kossinets and Watts 2009). These traits seem, from the collected data, to have different degrees of priority for e-Arabs, with ethnicity given top priority when forming connections. Once connections of shared ethnicity are formed, e-Arabs seek other characteristics, thus resulting in the formation of homophilous sub-groups within an umbrella ethnicity-dominated group. Therefore, ethnicity can be considered to act as a unifying bond that prevails over internal fragmentations among e-Arabs due to various other characteristics. This is especially the case when their ethnicity is attacked; put simply, although e-Arabs can be fragmented when against one another, they are united when against non-Arabs (cf. Boullata 1999). One interviewee pointed out that ‘despite the fragmentation, disputes, and tensions within the confines of Arab society, Arab tweeters are ordinarily highly skilled in uniting to demolish and knock down any external attempt to diminish or criticise Arab culture’ (I-17). This online characteristic of Arabs seems prevalent to a similar extent offline, where the values of pan-Arabism and Arab nationalism have deep roots (mark: e-pan-Arabism) (Kramer 1993).

### 3.2. E-Arabs versus Others

#### 3.2.1. E-Arabs Accusing Others

It is apparent from the data that Twitter-based accusations take various forms. For instance, online, one may accuse others of breaching the social contract (mark: social e-contract). Many Arab tweeters demand ‘parental care from their governments’ (I-14), such as demanding from the government free land, financial gifts on national days, doubled salaries in celebration of holy months, and the settlement of their loans (OC-3). They believe such treatment to be a component of the social contract. This online characteristic is prevalent to a similar extent offline, which is where the social contract is enforced (Thompson 2019). Another practice among e-Arabs consists of accusing others of corruption and untrustworthiness (mark: e-conflict). Reporting false corruption is commonplace in the Arab Twitterverse and ‘is applied by some tweeters to mess around, have a wild time, or gain attention and, therefore, attract more followers’ (F-4). Another issue is the criticism of others’ trustworthiness, which may involve accusing them of being hired or appointed through corruption (OC-6). This online characteristic is prevalent to a similar extent offline and can be attributed, at least in part, to limited regulation against false accusations. While paranoid personality disorder is a psychological issue in non-Arab cultures, it constitutes a social phenomenon in Arab culture, wherein Arabs are encouraged to resort to ethical as well as unethical ways of expressing their inescapable, established suspiciousness and mistrust of others. 

#### 3.2.2. E-Arabs Criticising Others

It is found that, in the Arab Twittersphere, there are various forms of criticism. To illustrate, online, one may criticise others’ tribes and families (mark: e-tribalism). This criticism will more than likely result in a fight, given that criticising a tribe is often perceived as ‘an insult to every member of the tribe’ (I-18). This online characteristic is prevalent to a similar extent offline, where criticism is usually not directed at individuals but rather at their tribes (Maisel 2018). Another form of criticism is directed towards others’ culturally unacceptable deeds (mark: e-collectivism). For instance, ‘if a woman expresses herself freely or shows some kind of openness, she, at times, encounters a societal attack to turn her back to her rightness’ (I-9). Online, one may intervene in others’ affairs (mark: e-collectivism) to the extent that, in the Arab Twitterverse, ‘everyone is terrified of everyone else’s gaze’ (I-28). Online, one may direct others (mark: e-collectivism). That is, Arab tweeters tend to ‘give advice and guidance to each other in all areas of life, as all feel in charge of all’ (F-2); moreover, they ‘interchangeably direct and are directed constantly and act reciprocally as mentors and mentees all the time, turning Twitter into an intensively instructive “therapy centre” crammed with directive tweets’ (I-19). These online characteristics are prevalent to a similar extent offline, as reflected in the presence of social monitoring, social unity, and the tendency to feel responsible for one another in Arab society. Arab society is better described as an ‘advice society’ (I-33), whereby one gives unsolicited advice to others on their private and public lives.

#### 3.2.3. E-Arabs Creating Conflict with Others

There is consistent evidence from the data that demonstrates that Twitter has opened a new chapter of polyvalent conflict between Arabs, facilitated by prejudice and discrimination (mark: e-prejudice). The act of telling jokes about race, nationality, tribe, ethnicity, religious schools of thought, pronunciation, dialects, and sects is an integral component of online Arab culture, triggering hostility among Arab tweeters (OI-34). This online characteristic is prevalent to a similar extent offline and can be attributed, at least in part, to the limited nature of the laws against prejudice and discrimination.

### 3.3. E-Arabs pro Themselves

#### 3.3.1. E-Arabs and Self-Marketing

Many e-Arabs are found to employ various strategies to increase their influence, social presence, and follower counts, for example, by creating multiple accounts (mark: e-manoeuvring). An individual may create a plethora of accounts with a variety of fake names, making all of them follow their main account to swell the ranks of their followers (OI-30). This online characteristic is prevalent to a similar extent offline, with the offline counterpart of childrearing, where having multiple children is a way of gaining status and feeling powerful within one’s social circle.

Tweeters may ‘buy’ followers or solicit undeserved retweets (mark: e-manoeuvring). Those who have created many accounts may ‘trade’ ‘followers’; in other words, one may pay another for all their fake accounts to follow one’s account (OI-2). Having more followers is a source of social supremacy, sovereignty, pride, and status, not only for individual tweeters but also for their whole tribe. By the same token, having a small number of followers denotes insult and humiliation. In the past, Arabs used to gain the title of ‘knight’ through their tribe, their spear, and their sword; in the present day, it is obtained through a sizeable social media presence and through the number of followers.

#### 3.3.2. E-Arabs and Self-Fabrication

The analysis revealed that many Arab tweeters employ various techniques to fabricate their public images. For example, online, one may exaggerate one’s achievements (mark: e-narcissism). Many Arab individuals (and institutions) tweet about their inconsequential, trivial, and minor achievements in an extravagant manner (F-41). At times, they celebrate ‘something out of nothing’ (I-34), such as ‘the celebration of obtaining their fake Ph.D. degrees’ (I-16). This online characteristic is prevalent to a similar extent offline, where the behaviour originates, with individuals seeking validation from others and striving to secure a positive public image by any means.

Online, one may praise oneself (mark: e-narcissism). If someone encounters a tweet that praises them, they will retweet and celebrate this praise, even if they know that the compliment is spurious and false (I-21). Likewise, online, one may grant oneself spurious titles (mark: e-narcissism). Some add such titles as ‘journalist’ or ‘photographer’ before their profile names, for instance, calling themselves ‘Poet Mohammed’ (OC-6). Many describe themselves as ‘researchers’, ‘analysts’, or ‘activists’, even though they have not undertaken any research, analysis, or campaigning. This online characteristic is prevalent to a similar extent offline, where self-aggrandisement is granted exclusively to the elite; accordingly, individuals (whether online or offline) are desperate for such accolades in order to associate themselves with that enviable group. Moreover, while narcissism is a psychological issue in non-Arab cultures, it forms a social phenomenon in Arab culture, where Arabs are encouraged to resort to ethical as well as unethical ways of expressing their narcissism.

Online, one may falsify knowledgeability (mark: e-manoeuvring). Some attend workshops merely to share photos from the event to share on Twitter, thereby feigning intellectual interest (I-17). Similarly, some open a random page of a book, place a coffee mug next to the page, take a picture, and post it on Twitter, giving the public a forged impression of their interest in reading (I-16). This online characteristic is prevalent to a similar extent offline, where knowledge is a feature of the elite; accordingly, individuals (whether online or offline) are desperate to display knowledge in order to associate themselves with a higher social class. 

#### 3.3.3. E-Arabs and Self-Pity

Some Arab tweeters employ tactics to engender public sympathy towards themselves, for example, by publicising one’s illness (mark: e-self-pity). An evergreen tweet is ‘pray for me’, to which is attached a photo of the tweeter visibly sick or showing some other difficulty (OI-4). There ensues a torrent of compassionate prayers from other tweeters, many of whom do not know the person (OC-7). Seen one way, this is an exploitation of the Twitter crowds to maximise prayers for oneself and, consequently, to ‘increase the likelihood of one’s recovery’ (I-17). This online characteristic is prevalent to a similar extent offline, where one publicises illness or death in the hope of collecting more prayers for victims. 

In online settings, social sympathy is created by sharing news of death (mark: e-self-pity). There is excessive tweeting about news of death, to the extent of ‘giving the impression that the entire Arab society will be shortly extinct’ (I-8). Sharing this type of news online is a means of using the social media masses to amplify prayers for the dead and, consequently, increase the probability of God’s forgiveness (F-3).

Online, social sympathy is created by showing generosity (mark: e-generosity). This online characteristic is prevalent to a similar extent offline, where the behaviour originates, with ‘givingness’ (a direct translation from Arabic), a deeply held, historical value that is widely appreciated in Arab society, being utilised as ‘the highway to social sovereignty, superiority, visibility, respect, prestige, power and honour’ (Hammadi 2007, pp. 156–57). Abdulrahman (1981) shows givingness as Arabs’ top personality trait. Al Zahrani (2002, p. 98) presents givingness as ‘the forefront of social values’. Al-Barghathi (2014, p. 13) states that ‘the Arab glorifies givingness, placing it above all other cultural values’. For Alwan (2019, p. 183), ‘perhaps the most prominent characteristic that the Arab is proud of and distinguishes them from others is givingness’. Under-givingness can even be socially treated like a criminal act for which one could be communally punished through means of social exclusion and marginalisation (Al-Sudais 1991; Hassani and Matloob 2019). A dearth of givingness can be a source of ‘defamation, blame and shame’ (Saleh 2012, p. 21).

## 4. Findings II (Offline Culture Being Counteracted Online)

This section demonstrates how offline culture may be counteracted online among Arabs, showing how online spaces may deviate from offline realities. It points out how online ethnic identity behaviour may not align with offline ethnic identity expectations.

### 4.1. E-Arabs Alongside Others

#### E-Arabs Empowering Others

The data analysis highlights multifaceted factors related to the Internet-based empowerment of individual Arabs. First, online, Arab women freely participate in the public domain (mark: e-participation), although offline, they are traditionally ‘associated with the domestic domain and protected by the concrete walls of homes’ (F-2). The Internet permits Arab women to access and proactively partake in the public domain while still within their domestic domain (OI-26). Second, online, the genders freely communicate (mark: e-communication across gender lines), although offline, communal customs (e.g., the social norm of gender separation) prevent such free communication (Crozier and Badawood 2009). The pseudo-anonymity of Twitter enables Arab users to bypass cultural customs by exchanging romantic posts and even adopting new sexual identities (OI-13). Third, online, one can freely express oneself emotionally and physically (mark: e-expression). Twitter enables a multitude of Arab women to express themselves not only through their words but also through their bodies in the sense that they share videos of themselves dressed and dancing provocatively while concealing their faces (I-24). Male e-Arabs are also sexually expressive, tweeting about sexual frustrations and defining themselves as, for example, ‘the Prince of Romance’ (OI-7), the ‘King of Love’ (OI-3), and the like. This online characteristic is less prevalent offline, where expression is controlled, and culturally defined gender expectations and roles are enforced (Bahoussi 2020).

### 4.2. E-Arabs versus Others

#### 4.2.1. E-Arabs Challenging the Elite

There was little doubt among the interviewees that Twitter challenges elitism in the Arab context in various ways. First, online, the elite is exposed (mark: e-exposure). Once an elite enters the Twitterverse, the members lose much of their status, ‘stepping outside their habitual shelter, being exposed to low-income individuals and disclosing their true shallow, superficial selves to the public’ (I-27). This online characteristic is less prevalent offline, where the elite is socially protected (Rayan 2019). Second, online, new elites emerge (mark: e-empowerment). Twitter has bred ‘the new elite’ (e.g., comedians and satirists), who are gradually undermining and replacing ‘the conventional elite’ (those with religious or managerial authority or with financial or academic capital). This online characteristic is less prevalent offline, where the means for social mobility are managed and controlled by the traditional elite (Rayan 2019). Third, online, innovation emanates from outside the elite circle (mark: e-empowerment). In the past, members of the conventional elite were the ones who coined terms and phrases; in the present day, the new elite has also taken on this role, even to the extent that the old elite has started to use their terms and phrases, which represents a significant shift in linguistic power relations (F-4). This online characteristic is less prevalent offline, where only the traditional elite possesses the capital to sponsor innovation (Murad 2019). Notably, this phenomenon also occurs outside of the Arab world, as the Internet has facilitated the global re-politicisation of the social fabric, thereby empowering individuals and enabling them to negotiate (or even try to balance) imbalanced power relations.

#### 4.2.2. E-Arabs Creating Conflict with Others

There is consistent evidence from the data that demonstrates that Twitter has created a new sphere where conflict between Arabs (OC-1) is created. First, online, one may provoke others (mark: e-conflict). Teasing GIFs, emojis, and exclamation marks are used excessively to provoke other tweeters (OC-5). Many Arab tweeters ‘scream’ at one another, as they have confidence in ‘the art of winning through a loud voice’ (I-15). This online characteristic is less prevalent offline, which could be explained by the fact that Arabic comes across as less friendly in written communication than when spoken (Hanafi 2012).

## 5. Findings III (Online Culture Being Enacted Offline)

This section demonstrates how online culture may be enacted offline among Arabs both in terms of how online ethnic identity behaviour may exert influence over offline ethnic identity expectations and how online spaces may shape offline realities.

### 5.1. E-Arabs versus Offline Arabs

#### E-Arabs Shaping Offline Culture

The boundary between the virtual and real worlds ‘has already collapsed because our offline self is brought into our online interactions, and our online interactions have changed how we interact offline’ (Marotta 2011, p. 460). One’s ethnic value systems can be modified by the blending of online and offline settings into one another, showing a sense of reciprocated inseparability (Albana 2014). Below is a collection of examples to illustrate this reciprocity.

Example 1 is that if cultural misconduct is undertaken in the Arabs’ physical world, they enthusiastically resort to social media to report it in ‘a blatant manner’ (F-1), complaining about and campaigning against it (mark: correction). In this case, social media functions as a ‘censorship authority capable of controlling offline society’ (F-1). Likewise, in consonance with some Arab authorities’ initiatives to combat corruption, some Arab tweeters energetically investigate corruption in the real world to then report it on Twitter (OC-60). The Arab online setting is, thus, being utilised to correct the Arab offline setting. Example 2 is that when some Arab employees start a fight on Twitter, they take it with them to their offline workplace, which means that Twitter can act as a means of intensifying conflict in Arabs’ offline spaces (mark: intensification). Example 3 is that the offline sense of tribalism has exerted influence over Twitter, which has, consequently, strengthened the offline sense of tribalism in various ways (cf. Al-Saggaf 2004) (mark: intensification). For instance, Twitter accounts are set up using tribal names. Moreover, tribal members connect through Twitter, although they may have never seen or even communicated with one another in offline situations. Poems, news, and details related to tribal glory are also proactively composed and cited. In this respect, offline ethnic ties may not simply passively go online but may actually become stronger, as members of an ethnic group can easily find their fellow members, communicate with them, establish online forums and groups, and seek sympathy and support. Example 4 is that, notwithstanding the limited presence of feminism in Arabs’ offline environs, feminism has a strong presence on Twitter (OC-1), which is enabling resistance to the male-dominated physical realm (mark: politicisation). Online, one may accuse others of being feminist, open, or free. Referring to an individual using these labels is considered to be ‘insulting and outrageous’ (I-15). The online domain has spread such concepts as feminism, freedom, and openness throughout the offline domain (Abu Hussein 2020).

Considering these examples, it could be said that to an extent, offline culture has been counteracted online among Arabs. This new online culture has then been enacted offline. Offline ethnic culture, at times, forms online ethnic culture, which, in turn, redefines offline ethnic culture (cf. Macfadyen et al. 2004). Phrased another way, the offline ethnic substance is *uploaded* and is shaped online, and then the reshaped ethnic substance is *downloaded* to come full circle and shape the offline ethnic substance (Panagakos 2003).

## 6. Concluding Remarks

### 6.1. Emerging Theory

This article has scrutinised the linkage between ethnicity and social media usage by examining how offline culture manifests itself online among Arabs. The offline and online ethnic characteristics of Arabs were drawn from the literature and from interviews and observations of Arab users of social media, respectively. These online and offline characteristics were then cross-referenced to reach three main findings: offline culture has been enacted online, offline culture has been counteracted online, and online culture has been enacted offline.

That said, Arab online ethnic culture was found to be more enactive and less counteractive of offline culture. In other words, the comparison between the online and offline ethnic characteristics of Arabs revealed many likenesses and few variances, indicating that Arabs’ online ethnic characteristics are largely derivative of or even mimic their offline ethnic characteristics. There appears to be no, or at least a highly porous, boundary between the virtual and real-life worlds of Arabs; most of their online activities stay rooted in their reality and are based on the same needs and concerns that drive their offline activities. 

The large influence of offline ethnicity over online behaviour presents unwelcome news for those who had placed their hopes in technostructural efforts to push (or even force) human society into a monolithic e-culture and homogenised e-ethnicity. Similarly, those who dreamt of an online ethnic culture without roots in the offline world and with members who sustain a unique language, discourse, clothing, appearance using avatars, etc., will not find comfort in these findings.

While online spaces seem to reinforce offline realities, online behaviour does not always align with offline ethnic identity expectations. The current study reconciles this discrepancy with the notion of ‘e-ethnic culture’, which acknowledges that ethnic activities online are largely extensions of an offline ethnic culture, but they are nonetheless distinct from this culture due to their digital environment, which gives rise to deviations (cf. Anishchenkova 2014). Moreover, such an e-ethnic culture implies a digital version of the Arab, namely the ‘e-Arab’. This is why the article is titled ‘The Culture of e-Arabs’. This study finds that Arabs and e-Arabs are largely alike, yet not entirely.

It is plausible that online, some people embrace an ethnicity that is not theirs in offline settings. The high level of anonymity online may encourage ‘falsified ethnicity’ (cf. Fanon 1970). A falsified online ethnicity could allow one to break free from ethnicity-related constraints that they experience in real life. Relatedly, it is beyond the scope of the current research to examine whether individuals with mixed ethnicity (e.g., the child of an Arab and a Caucasian) may, intentionally or subconsciously, shift between their various ethnicities to match the ethnicity of those with whom they are communicating online.

### 6.2. Limitations

This study used a non-participant observation method to research individual tweeters’ activity on Twitter. A limitation of this method is that placing a subject under observation, specifically when they are aware of the observation, can result in the controlling or even modification of their behavioural patterns, which may impact the results obtained. While this limitation can neither be methodologically nor ethically eliminated, an effort was made to minimise it through various techniques. One such technique was to have the observation last for an average of four continuous hours, with the continuity and length of the observation intended to provide the observee with greater opportunity to behave naturally while under observation. The authors thought that the awareness of the observation or, at least, conscious modification of behaviour would reduce over time. The second technique was that the data from one observee was checked against the data from other observees; moreover, the data from this data collection method were checked against the data from the other methods. The third technique was that the data from this method were not applied as a principal source but, instead, were used to enrich the data gathered through the other methods.

A notable methodological limitation in this study is the inability to guarantee that a participant in the collective observations was indeed an Arab. That said, the last name of one’s profile name could help identify whether one was an Arab, although it is still possible for someone to falsify this. As for the individual observations, focus groups, and individual interviews, all participants were asked as part of the informed consent to confirm that they were Arab.

It was not technically possible to identify the locations of the participants in the collective observations, which constitutes a methodological limitation. However, the locations of the participants in the individual observations, focus groups, and individual interviews were diverse, spanning all Arab countries except for Comoros, Djibouti, Mauritania, and Somalia. It should be remarked that the data are slightly skewed towards Egypt, Jordan, Saudi Arabia, and Lebanon, as most of the participants came from these countries. Notably, these countries tend to exert the most cultural influence over Arab society.

As for its spatial limitations, this study examined Arabs’ behaviour exclusively on Twitter, while other social media platforms, whether owing to their differing structure or community, could elicit different behaviour. As for its ethnic limitations, this study focused on Arabs, thereby limiting the applicability of the findings and theoretical propositions drawn from this study to other ethnic groups.

A significant methodological limitation of this study, owing to its use of the maximum variation sampling technique, is the lack of focus with regard to the sample. Given the qualitative nature of the study, this is not to say that the findings need to be quantified through further research to be generalisable. Moreover, any ethnic culture, be it online or offline, is multidimensional and enormous, so much so that both spatially large-scale and temporally continuous efforts are required to achieve a comprehensive level of documentation. The current study is neither large-scale nor continuous, and further research is, thus, required to strengthen (or, rather, problematise) the documented configurations.

That said, a strong point of the current study is that it acts as a ‘start-up’, establishing an initial conceptual framework for future studies on various online ethnic cultures. This study could be the seed from which grows what could be called ‘e-ethnic studies’, a field dedicated to theorising about the role that the ethnic factor should play in the academic investigation of the online landscape. This field would promote the conceptualisation of the digital world as a cultural context that entails ethnically situated norms, values, and capabilities (Hine 2013). It should regard the online realm as an ‘ideological construct’ (Varis 2016, p. 58) composed of ethnic-specific appropriations and meanings.

## Figures and Tables

**Table 1:** Data analysis of interviews and observations to identify whether offline culture is enacted or counteracted online among Arabs.

Mapping the Online Characteristics of Arabs						
Macro Vision	Meso Vision	Micro Vision	Mark	Source	Translated Quotes from the Raw Data	Quotes from the Raw Data in Their Original Language
Offline Culture Enacted Online	E-Arabs alongside Others	E-Arabs Empowering Others	E-identity (concealing one’s identity)	Observation	‘Many users hide their identity, making it difficult to know whether they are men or women’.	“يخفي العديد من المستخدمين هويتهم ، مما يجعل من الصعب معرفة ما إذا كانوا رجالًا أم نساء”.
		E-Arabs Honouring the Present	E-familialism (relatives supporting each other)	Observation	‘People on Twitter support their relatives’.	“الأشخاص على التويتر يدعمون أقاربهم”.
			E-pan-Arabism (Arabs supporting each other)	Interview	‘Arab unity against any external attack is usually strong on Twitter’.	“الوحدة العربية ضد أي هجوم خارجي عادة ما تكون قوية على تويتر”.
			E-flattery (showing loyalty to important individuals)	Interview	‘Users respect and show excessive respect for VIPs in order to gain interests through them’.	“يحترم المستخدمون الشخصيات المهمة ويظهرون الاحترام المفرط لكسب المصالح من خلالهم”.
	E-Arabs v. Others	E-Arabs Accusing Others	E-conflict (accusing others of corruption and untrustworthiness)	Interview	‘Criticising others for corruption has become a staple of Twitter’.	“أصبح انتقاد الآخرين بسبب الفساد عنصرًا أساسيًا في التويتر”.
			Social e-contract (accusing others of breaching the social contract)	Interview	‘Many of the criticisms indicate that many of the expectations for the authorities in their paternalistic roles were not fulfilled’.	“تشير العديد من الانتقادات إلى أن العديد من التوقعات من السلطات في دورها الأبوي لم تتحقق”.
		E-Arabs Criticising Others	E-tribalism (critisising others’ tribes and families)	Interview	‘Sense of tribe has a clear presence; There are critics and defenders of tribes’.	“الشعور بالقبيلة له حضور واضح ؛ هناك نقاد ومدافعون عن القبائل.
			E-collectivism (critisising others’ culturally unacceptable deeds)	Interview	‘People feel a sense of guardianship over one another, which is a bother to me’.	“يشعر الناس بإحساس الوصاية على بعضهم البعض ، وهذا يزعجني”.
			E-collectivism (intervening in others’ affairs)	Interview	‘Everyone makes themselves responsible for everyone else’.	“كل شخص يجعل نفسه مسؤولاً عن أي شخص آخر”.
			E-collectivism (directing others)	Interview	‘People on Twitter love to advise one another’.	“يحب الأشخاص على التويتر تقديم المشورة لبعضهم البعض”.
		E-Arabs Creating Conflict with Others	E-prejudice (prevalent prejudice and discrimination)	Interview	‘You get all kinds of bias online’.	“يمكنك الحصول على جميع أنواع التحيز عبر الإنترنت”.
	E-Arabs pro Themselves	E-Arabs and Self-marketing	E-manoeuvring (increasing one’s influence by creating multiple accounts)	Interview	‘You see people who have more than one account’.	“ترى شخصًا لديه أكثر من حساب”.
			E-manoeuvring (increasing one’s influence by buying followers or soliciting undeserved retweets)	Interview	‘I know many people who buy followers’.	“أعرف الكثير من الأشخاص الذين يشترون المتابعين”.
			E-visibility (increasing one’s influence by ensuring a social presence)	Interview	‘One wants a presence on Twitter, even if one just writes “Good morning” or “Blessed Friday”’.	“يريد الشخص أن يكون له حضور على التويتر، حتى لو كتب مجرد "صباح الخير "أو "جمعة مباركة”.
			E-visibility (increasing one’s influence by drawing others’ attention)	Interview	‘People set up prizes to attract attention and become famous’.	“يضع الناس جوائز لجذب الانتباه وليصبحوا مشهورين”.
		E-Arabs and Self-fabrication	E-narcissism (exaggerating one’s achievements)	Interview	‘Exaggeration of accomplishments is a terrible thing on Twitter’.	“المبالغة في الإنجازات أمر مروع على التويتر”.
			E-narcissism (praising oneself)	Interview	‘I am surprised at how many people constantly praise themselves’.	“أنا مندهش من هؤلاء الأشخاص الذين يثنون على أنفسهم باستمرار”.
			E-narcissism (granting oneself spurious titles)	Interview	‘He calls himself an expert, and another calls herself a reporter’.	“هذا يسمي نفسه خبير ، وآخر يطلق على نفسه إخباري”.
			E-manoeuvring (falsifying knowledgeability)	Interview	‘Many people represent themselves as someone who is an expert, loves knowledge, and actively attend training courses’.	“كثير من الناس يمثلون أنفسهم كشخص خبير، أو يحب المعرفة أو أنه يحضر دورات تدريبية باستمرار لتطوير مهاراته”.
		E-Arabs and Self-pity	E-self-pity (creating social sympathy by putting on a show of illness)	Interview	‘The funny thing is that he puts up a picture of himself when he is sick and writes: “Pray for my recovery”’.	“الشيء المضحك هو أنه وضع صورة لنفسه وهو مريض وكتب: "ادعوا لي بالشفاء”.
			E-self-pity (creating social sympathy by sharing news of death)	Interview	‘One feels that most humans have died because of the huge amount of news of death on Twitter’.	“ممكن الفرد يخيل إليه أن معظم البشر قد ماتوا بسبب الكم الهائل من الأخبار الميتة على تويتر”.
			E-generosity (creating social sympathy by showing generosity)	Interview	‘Showing pride in generosity and kindness is common among Arab tweeters’.	“الاعتزاز بالكرم والعطف شائع بين المغردين العرب”.
Offline Culture Counteracted Online	E-Arabs Alongside Others	E-Arabs Empowering Others	E-participation (women participating freely in the public domain)	Observation	‘There is a clear presence of women’s participation, to the extent that there appears to be almost one entry by a woman for each post by a man’.	“هناك حضور واضح لمشاركة المرأة، لدرجة أنه يبدو أن هناك مشاركة واحدة تقريبًا من قبل امرأة لكل مشاركة بواسطة رجل”.
			E-contact across gender lines (genders freely communicating)	Observation	‘Men and women comment on each other’s entries and interact with each other’.	“الرجال والنساء يعلقون على تعليقات بعضهم البعض ويتفاعلون مع بعضهم البعض”.
			E-expression (freely expressing oneself emotionally and physically)	Observation	‘Men and women express themselves emotionally as well as share body images and videos’.	“الرجال والنساء يعبرون عن أنفسهم عاطفياً وكذلك يشاركون صور مقاطع لأجسادهم”.
	E-Arabs v. Others	E-Arabs Challenging the Elite	E-exposure (exposing the elite)	Interview	‘The Internet has undoubtedly dropped the prestige accorded to the elites in the real world’.	“لقد أسقط الإنترنت بلا شك المكانة الممنوحة للقروبات النخبوية في العالم الحقيقي”.
			E-empowerment (new elites are emerging)	Interview	‘There are new types of elites appearing in social networks that have no roots in real life’.	“هناك أنواع جديدة من النخب تظهر في الشبكات الاجتماعية وليس لها جذور في الحياة الواقعية”.
			E-empowerment (innovation emanating from outside the elite circle)	Interview	‘Hobbies and innovations emerged from individuals who do not belong to the elites that we are accustomed to … The Internet has opened the door for everyone…’.	“ظهرت هوايات وابتكارات من أفراد لا ينتمون إلى النخب التي اعتدنا عليها … لقد فتح الإنترنت الباب للجميع …”.
		E-Arabs Conflicting with Others	E-conflict (accusing others of being open, free, or feminist)	Interview	‘Social media networks have opened the way for people to bicker and accuse each other of openness.’	“لقد فتحت شبكات التواصل الاجتماعي الطريق أمام الناس للتصارع واتهام بعضهم البعض بالانفتاح”.
			E-conflict (provoking others)	Interview	‘People unnaturally tease and provoke one another’.	“الناس يضايقون ويستفزون بعضهم البعض بشكل غير طبيعي”.
Online Culture Enacted Offline	E-Arabs v. Others	E-Arabs Shaping Offline Arabs	Correction (using social media to correct offline behaviour)	Interview	‘Social media has become like the religious police in offline life’.	“أصبحت شبكات التواصل الاجتماعي أشبه لهيئة الأمر بالمعروف والنهي عن المنكر”.
			Intensification (using social media to encourage certain behaviours, values, and norms in offline settings)	Interview	‘My colleague brings our disagreement online to our workplace’.	“زميلي في العمل يجلب اختلافنا على شبكات التواصل الاجتماعي معه إلى العمل”
			Politicisation (using social media to politicise offline domains)	Interview	‘Feminism has entered our life because of social networks’.	“النسوية دخلت حياتنا بسبب شبكات التواصل الاجتماعي”

## Data Availability

Not available.
